# The Effects of Conflicts and Self-Reported Insecurity on Maternal Healthcare Utilisation and Children Health Outcomes in the Democratic Republic of Congo (DRC)

**DOI:** 10.3390/healthcare9070842

**Published:** 2021-07-03

**Authors:** Tingkai Zhang, Xinran Qi, Qiwei He, Jiayi Hee, Rie Takesue, Yan Yan, Kun Tang

**Affiliations:** 1 Vanke School of Public Health, Tsinghua University, Beijing 100084, China; ztk17@mails.tsinghua.edu.cn (T.Z.); qixinran@ccmu.edu.cn (X.Q.); heqiwei@mail.tsinghua.edu.cn (Q.H.); SinoScriptshare@gmail.com (Y.Y.); 2 School of Life Sciences, Tsinghua University, Beijing 100084, China; 3 School of Nursing, Capital Medical University, Beijing 100069, China; 4 School of Medicine, Tsinghua University, Beijing 100084, China; 5 School of Public Health, Faculty of Medicine, The University of Queensland, Brisbane 4006, Australia; j.hee@uqconnect.edu.au; 6 Health Section Programme Division, UNICEF Headquarters, New York, NY 10017, USA; rtakesue@unicef.org

**Keywords:** maternal health-seeking behaviours, childhood diseases, armed conflict, the Democratic Republic of the Congo (DRC)

## Abstract

***Background*** The Democratic Republic of Congo (DRC) has experienced political unrest, civil insecurity, and military disputes, resulting in extreme poverty and a severely impaired health care system. To reduce the morbidity and mortality in women and children by strengthening healthcare, this study aimed at exploring the relationship between self-reported insecurity of mothers and maternal health-seeking behaviours and diseases in children in the DRC. ***Method*** Data collected from 8144 mothers and 14,403 children from the Multiple Indicators Cluster Survey (MICS) conducted by the National Institute of Statistics in 2017–2018, in collaboration with the United Nations Children’s Fund (UNICEF), was used. The severity of the conflict in different provinces was measured using the Uppsala Conflict Data Program (UCDP) reports. Multivariate logistic regression and stratified analysis were utilized to explore the association between conflicts with maternal health-seeking behaviours and diseases among children. ***Results*** High self-reported insecurity was positively associated with skilled antenatal care (OR1.93, 95%CI 1.50–2.49), skilled attendants at delivery (OR1.42, 95%CI 1.08–1.87), and early initiation of breastfeeding (OR1.32, 95%CI 1.04–1.68). These associations were more significant in regions with more armed conflict. It was also found that children of mothers with high self-reported insecurity were more likely to suffer from diarrhoea (OR1.47, 95%CI: 1.14–1.88), fever (OR1.23, 95%CI 1.01–1.50), cough (OR1.45, 95%CI 1.19–1.77), and dyspnea (OR2.04, 95%CI 1.52–2.73), than children of mothers with low self-reported insecurity. ***Conclusions*** Conflicts increases mothers’ insecurities and negatively affects children’s development. However, high conflict regions have to increase governmental and international assistance to promote the availability and access to maternal and child health services.

## 1. Introduction

Globally, approximately 2 billion people live in conflict-plagued areas, where they experience violence, diseases, and malnutrition [1], contributing to 30% to 50% of the global maternal and child deaths [2]. In particular, conflict-affected low- and middle-income countries (LMICs) have been associated with poorer maternal and child health (MCH) outcomes [3]. It was reported that out of the 34 LMICs furthest from achieving global MCH targets, 22 were identified as conflict-stricken countries [4].

The United Nations (UN) Sustainable Development Goals (SDGs) [5,6], a goal of which is to improve maternal and children’s health through increased financing, strengthened policies and improved onsite services [7,8], have reported that efforts were hindered by the presence of conflict, indicating that violence and instability can threaten governmental and international aid, further deterring health promotion.

Since 1960, after the Democratic Republic of Congo (DRC) gained independence, the country has been plagued by civil unrest and political instability [9]. Continuous conflict and violence particularly in the eastern provinces between local militia and government forces resulted in frequent mass displacement [9,10,11,12]. Approximately 1.7 million new displacements occurred in 2019 and 1.4 million new displacements occurred in early 2020, the majority of which were women and children [13]. Women and children in the DRC lack fundamental physiological and safety needs, resulting in feelings of anxiety and insecurity [14]. The conflicts between the communities also exist due to insufficient resources such as food and water [14]. The conflicts and violence have impaired the development of the DRC, leaving the DRC undeveloped in many areas such as housing, transport, education, and more importantly, essential healthcare [15,16]. The war-ravaged DRC needs significant humanitarian support for fundamental life supplies as millions of people to lack access to essential health care services. However, with limited help from humanitarian responders, the mass displacement severely hinders the establishment of healthcare facilities and results in extreme malnutrition and poverty [17], as well as high maternal and child mortality rates in the DRC [18].

As reported from previous studies, conflict-affected regions often present with major health concerns, especially in maternal and child healthcare [19]. In the DRC, the maternal mortality ratio (MMR) was 473 deaths per 100,000 live births in 2017 and the children under-five mortality ratio was 8480 deaths per 100,000 live births in 2019 [14]. Disparities in maternal and child health services, including antenatal care (ANC), having a skilled attendant at delivery, post-natal care (PNC), and children’s welfare checks, exist between different provinces in the DRC [19,20].

To date, few studies have reported on how political instability affects maternal and child healthcare, especially on the utilisation of maternal and child healthcare in the conflict-affected DRC, which gives rise to the need to investigate how various determinants and factors affect maternal and children’s health. In this study, we assessed the association of the severity of conflicts and self-reported insecurity with healthcare utilisation between regions in the DRC. We aim to provide decision-makers with comparative information on the effects of conflicts on maternal and child healthcare utilisation and to provide practical and evidence-based guidance to inform future health interventions and policies.

## 2. Methods

### 2.1. Participants

In this study, we utilised data from the 2018 Multiple Indicator Cluster Survey (MICS), which presents more than 150 key indicators, providing information on women and children in the areas of health, income, nutritional statuses, and education in the DRC. A total of 8144 mothers aged 15 to 49 years old and 14,403 children under five were included in this study. The 2018 MICS consists of surveys conducted by the National Institute of Statistics in the DRC, a public institution governed by the Ministry of Planning in collaboration with the United Nations International Children’s Emergency Fund (UNICEF) and the United States Agency for International Development (USAID). The survey was conducted from 2017–2018. Stratified random sampling was used for collecting data. Except for Kinshasa, the other 25 provinces of the DRC were respectively divided into three strata (statutory cities stratum/cities stratum/chiefdom stratum). 76 strata were defined and then a total of 721 clusters were systematically selected with probability proportional to the size. After establishing the household list of the selected cluster, the system samples of 30 households were extracted in each cluster, respectively (21,630 households in total). Seven questionnaires were required for each household. The questionnaires covered multiple indicators of various epidemics, sexual and reproductive health, mental health, and water, sanitation and hygiene, etc. The fieldwork teams were trained for at least four weeks and deployed to sampled clusters of households. Interviewers used tablet computers to conduct face-to-face interviews with eligible respondents. The details of data collection methods were described elsewhere [20,21].

To measure the severity of conflicts in each province of the DRC, data from the Uppsala Conflict Data Program (UCDP) was used. The UCDP is a leading global provider of data on organized violence. The main sources of conflict records of UCDP included global newswire reporting, global monitoring and translation of local news by British Broadcasting Corporation (BBC), and secondary sources [22,23]. For this study, data from the UCDP Georeferenced Event Dataset (GED) Global version 20.1 [22,23] was used for the provision of data on conflict. The basic unit of analysis of the UCDP GED data set is the “event” of conflicts. An event of conflict is defined as an individual incident in which an organised actor uses armed forces against another organised actor or civilian, causing at least one direct death at a specific place and date [22,23]. In this study, we used the definition of UCDP GED as a reference. Events of conflict were sufficiently detailed enough to be geo-coded down to the village level, making data extraction on individual DRC provinces achievable.

### 2.2. Measures and Variables

#### 2.2.1. Self-Reported Insecurity

The variable ‘self-reported insecurity (Low insecurity/Medium insecurity/High insecurity)’ was used to estimate the level of insecurity felt by each participating mother and to evaluate the severity of conflicts at the community level. In the original questionnaire this question was described as “How safe do you feel walking alone in your neighbourhood after dark?” with the options ‘very safe’, ‘safe’, ‘unsafe’, ‘very unsafe’, and ‘never walk alone after dark’. ‘Very safe’ and ‘safe’ was recoded to ‘low insecurity’, ‘unsafe’ to ‘medium insecurity’, and ‘very unsafe’ and ‘never walk alone after dark’ to ‘high insecurity’.

#### 2.2.2. The Severity of Conflicts

To study the associations between conflicts and maternal and child health at both the community level and sub-regional level, a variable ‘the severity of conflicts’ was created. All conflicts records were obtained from UCDP. To measure the severity of conflicts before and during the MICS6 survey (2017–2018) in the DRC, battled related deaths (BRDs) for every province from 2016 to 2018 were added. 2016–2018 cumulative BRDs were then standardized according to the population of each province. The standardized death rate (per million people) was used to classify ‘the severity of conflicts’ in each province. The variable “the severity of conflicts” was categorized into low severity (less than 13 BRDs per 1,000,000 persons) and high severity (more than 13 BRDs per 1,000,000 persons, using the median of BRD as the cut-off).

#### 2.2.3. Maternal Health-Seeking Behaviours

Multiple maternal health-seeking behaviours were part of our focused outcomes, which included seeking professional ANC more than four times, having skilled attendants at delivery, receiving PNC, and early initiation of breastfeeding (EIB). Receiving professional ANC more than four times referred to mothers who have seen health professionals (doctors, nurses, midwives, or other qualified professionals) more than four times for ANC during pregnancy. Having skilled attendants at delivery referred to mothers who have had health professionals (doctors, nurses, midwives, or other qualified professionals) to assist with the delivery. Receiving PNC referred to any PNC within 48 h after delivery while in the healthcare facility or at home. EIB was defined as newborns who were breastfed within an hour after their birth. All four maternal health-seeking behaviours were defined according to World Health Organisation (WHO) indicators [24].

#### 2.2.4. Diseases of Children under Five

The prevalence of various diseases in children was another outcome investigated. Four diseases—diarrhoea, fever, cough, and dyspnea—were included in this study. All four variables were dichotomous and referred to whether the child (under five years of age) had suffered from any of the above in the past two weeks.

#### 2.2.5. Other Covariates

Demographic and socio-economic characteristics of mothers and children were considered as covariates in this study. Maternal characteristics included age, region (rural/urban), household economic status (quintile wealth index), and maternal education level (preschool/primary school/lower secondary school/higher secondary school/above). The characteristics of children included age and sex.

### 2.3. Statistical Analysis

Descriptive statistics were conducted on the prevalence of the severity of conflicts and self-reported insecurity in 26 DRC provinces. The distribution of maternal and children demographic and socio-economic characteristics, multiple maternal health-seeking behaviours, and diseases in children were illustrated among groups of women with different levels of self-reported insecurity, using descriptive analyses.

The association between self-reported insecurity, maternal health-seeking behaviours, and diseases in children was analysed using multivariate logistic regression modelling. The model for maternal health-seeking behaviours was adjusted for maternal age, region, household economic status, maternal education level, and the severity of conflicts. The model for diseases in children was adjusted for maternal age, region, household economic status, maternal education level, child age, child sex, and the severity of conflicts. Stratified analysis was then adopted to further explore the associations between self-reported insecurity, multiple maternal health-seeking behaviours, and diseases in children under different severity of conflict. Multivariate logistic regression models with the same covariates as described above were used in each stratified analysis. Results were reported as odds ratios (ORs) and 95% confidence intervals (95%CI). The level of statistical significance was set at 5% (*p* < 0.05) for all statistical analyses. All statistical analyses were performed using Stata/SE version 16.0 (StataCorp LLC, College Station, TX, USA).

## 3. Results

### 3.1. Sample Characteristics

#### 3.1.1. The Severity of Conflicts

**Figure 1:**
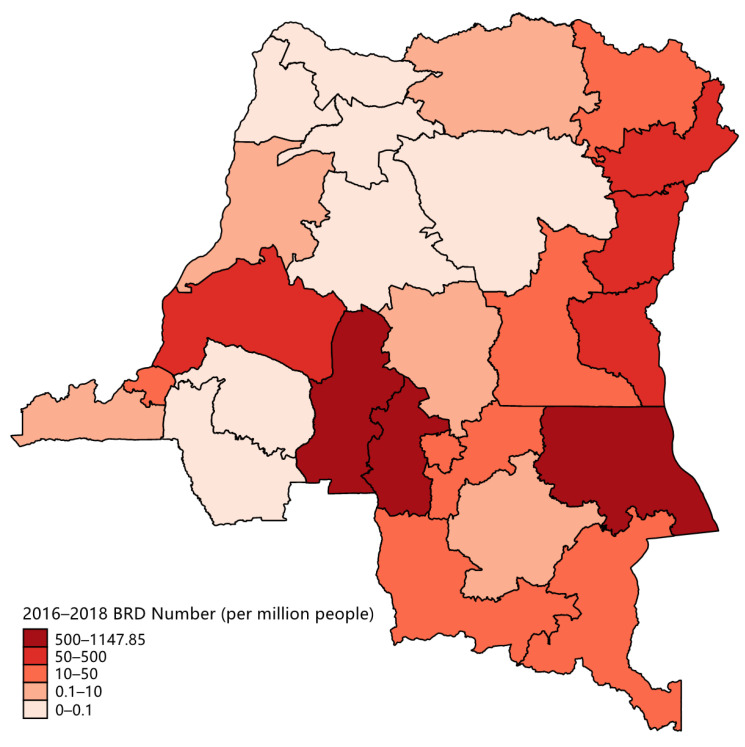
Different levels of conflict severity in each province in the DRC.

Table 1 presents the 2016–2018 cumulative BRDs and standardized death rate (per million people) of each province in the DRC. Figure 1 presents the 2016–2018 cumulative BRD number (per million people) in each province in the DRC, indicating the severity of conflicts in each province.

#### 3.1.2. The Self-Reported Insecurity of Females in the DRC

**Figure 2:**
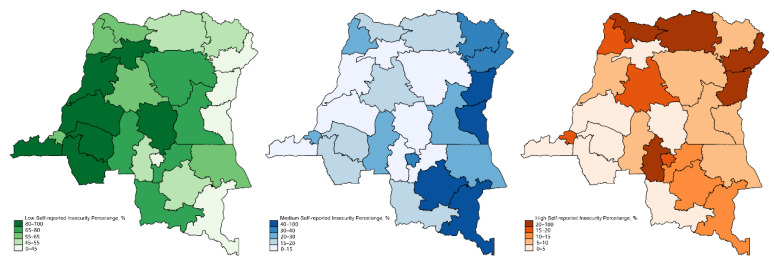
Distribution of different levels of self-reported insecurity in provinces of females in the DRC †. † Green: Low self-reported insecurity; Blue: Medium self-reported insecurity; Orange: High self-reported insecurity.

Figure 2 presents the distribution of different self-reported insecurity levels of females in each province in the DRC. We found that all females living in the DRC generally presented with some level of insecurity. In most areas, a mixture of low, medium, and high self-reported insecurity can be observed. Nord Kivu, Sud Kivu, Haut Lomami, and Haut Katanga had the highest prevalence of medium self-reported insecurity, and Bas Uele, Nord Ubangi, and Kasai Central province had the highest prevalence of high self-reported insecurity.

The provinces with a higher proportion of mothers with medium levels of insecurity are mainly located in the east and southeast regions of the DRC. The provinces with a higher proportion of mothers with high levels of insecurity are mainly located in the north and northeast regions of the DRC.

#### 3.1.3. Maternal Health-Seeking Behaviours and Diseases in Children

Table 2 and Table 3 present the demographic and socio-economic characteristics of the participating 8144 mothers and 14,403 children. Overall, 34.28% of mothers lived in rural areas, and the remaining 65.72% lived in urban areas, with 18.74% of mothers having attended preschool, 35.98% attended primary school, 14.59% attended lower secondary school, 27.68% attended higher secondary school and 3.00% attended other forms of education. Overall, 11.30% of females were from a wealthy household, and 25.98% of females were from a poor household, and 62.75% of females lived in regions with severe conflicts and 37.25% of females lived in areas with minor conflicts. Overall, 28.62% of females reported a moderate level of insecurity, and 12.26% reported higher-level insecurity in their daily lives. As expected, females living in regions with severe conflict reported higher levels of insecurity.

Overall, although 84.26% of mothers reported having a skilled attendant at delivery, less than half reported receiving adequate maternal health services (ANC4+, 41.52%, and PNC, 6.56%). When stratified by levels of self-reported insecurity, only 54.06% of mothers that reported high levels of insecurity had received ANC more than four times (44.86% for mothers with medium self-reported insecurity, 37.31% for mothers with low self-reported insecurity) and only 3.94% of them received PNC. However, a high percentage of mothers that reported high levels of insecurity had skilled attendants at delivery (87.53%) and EIB (53.26%).

The characteristics of the 14,403 children are presented in Table 3. The majority of children lives in rural areas (65.93%) and regions with high conflict (64.45%). Fever was the most prevalent in children under five (28.01%), followed by cough (26.33%), diarrhoea (15.75%), and dyspnea (9.36%). When stratified by levels of mothers’ self-reported insecurity, an increasing trend in the prevalence of diseases in children can be seen with increasing levels of self-reported insecurity. For example, fever prevalence in children of mothers who reported low, medium, and high levels of self-reported insecurity were 27.06%, 28.69%, and 30.98%, respectively.

### 3.2. Maternal Health-Seeking Behaviours

Table 4 presents the associations between self-reported insecurity with multiple maternal health-seeking behaviours. Compared to mothers with low levels of self-reported insecurity, mothers in the DRC with medium levels of self-reported insecurity and high levels of self-reported insecurity were more likely to seek professional ANC and have skilled attendants during delivery. Dose effect was observed in this analysis, indicating that as the level of self-reported insecurity increases, maternal health-seeking behaviours also increases. Using mothers with low levels of self-reported insecurity as a reference, the association between mothers who reported high levels of insecurity with ANC (OR: 1.93, 95%CI: 1.50–2.49) and having a skilled attendant at delivery (OR: 1.42, 95%CI: 1.08–1.87) were found to be significantly higher than the association between mothers who reported moderate levels of insecurity with ANC (OR: 1.30, 95%CI: 1.07–1.58) and having a skilled attendant at delivery (OR: 1.28, 95%CI: 1.02–1.62). Furthermore, when compared to mothers who reported low levels of insecurity, mothers who reported high levels of insecurity were more inclined to adopt EIB after delivery (OR: 1.32, 95%CI: 1.04–1.68) and mothers who reported moderate levels of insecurity were more likely to seek PNC (OR: 1.55, 95%CI: 1.12–2.16).

### 3.3. Diseases in Children under Five

The associations between self-reported insecurity with diseases in children are presented in Table 5. Using low levels of self-reported insecurity as a reference, high levels of self-reported insecurity was found to be significantly associated with diarrhoea (OR: 1.47, 95%CI: 1.14–1.88), fever (OR: 1.23, 95%CI: 1.01–1.50), cough (OR: 1.45, 95%CI: 1.19–1.77), and dyspnea (OR: 2.04, 95%CI: 1.52–2.73), and moderate levels of insecurity was found to be significantly associated with diarrhoea (OR: 1.45, 95%CI: 1.21–1.74) and dyspnea (OR: 1.68, 95%CI: 1.36–2.08). Children of mothers who reported high levels of insecurity were more likely to have diarrhoea and dyspnea than children of mothers who reported moderate levels of insecurity.

### 3.4. Stratification by the Severity of Conflicts

The associations between self-reported insecurity with maternal health-seeking behaviours, stratified by the severity of conflicts in the provinces, are presented in Table 6. In provinces with severe conflicts, positive associations were found between mothers who reported high levels of self-reported insecurity with ANC4+ (OR: 2.23, 95%CI: 1.64–3.03), skilled attendants at birth (OR: 1.91, 95%CI: 1.29–2.82), and EIB (OR: 1.63, 95%CI: 1.21–2.21). However, these mothers were also found to be significantly less likely to receive or seek PNC (OR: 0.39, 95%CI: 0.19–0.77). Positive associations were also found between mothers who reported moderate levels of self-reported insecurity with ANC4+ (OR: 1.40, 95%CI: 1.10–1.77), skilled attendants at delivery (OR: 1.77, 95%CI: 1.29–2.43), and EIB (OR: 1.32, 95%CI: 1.05–1.65). When stratified by the severity of the conflict, dose effects were observed, again indicating that as the level of self-reported insecurity increases, maternal health-seeking behaviours also increases. In provinces with low conflict severity, moderate levels of self-reported insecurity were found to be positively associated with receiving PNC (OR: 2.30, 95%CI: 1.51–3.50), but negatively associated with having skilled attendants at delivery (OR: 0.67, 95%CI: 0.50–0.89).

The associations between self-reported insecurity with diseases in children, stratified by the severity of conflicts in the provinces are presented in Table 7. In provinces with severe conflicts, positive associations were found between children of mothers who reported moderate and high levels of self-reported insecurity with diarrhoea (OR: 1.41, 95%CI: 1.13–1.75; and OR: 1.44, 95%CI: 1.06–1.95 respectively), and dyspnea (OR: 1.70, 95%CI: 1.31–2.21; and OR: 2.05, 95%CI 1.44–2.93 respectively) than children of mothers who reported low levels of self-reported insecurity. Positive associations were also found between children of mothers who reported high levels of self-reported insecurity with cough (OR: 1.47, 95% CI: 1.15–1.87) than children of mothers who reported low levels of self-reported insecurity.

In provinces with minor conflicts, positive associations were found between children of mothers who reported moderate levels of self-reported insecurity with diarrhoea (OR: 1.56, 95%CI: 1.14–2.14), fever (OR: 1.27, 95%CI: 1.01–1.61), cough (OR: 1.40, 95%CI: 1.10–1.77), and dyspnea (OR: 1.64, 95%CI: 1.17–2.29) than children of mothers who reported low levels of self-reported insecurity. Positive associations were also found between children of mothers who reported high levels of self-reported insecurity with dyspnea (OR: 1.91, 95%CI: 1.23–2.95) than children of mothers who reported low levels of self-reported insecurity.

## 4. Discussions

The result of our study suggests three significant findings. Firstly, mothers who reported moderate and high levels of self-reported insecurity have higher odds of having ANC, skilled attendants at delivery and EIB as compared to mothers who reported low levels of self-reported insecurity. Maternal health-seeking behaviours were found to increase as the level of self-reported insecurity increases. Secondly, when stratified by the severity of conflicts, mothers living in regions with severe conflicts were more likely to have ANC, skilled attendants at delivery and EIB than mothers living in regions with minor conflicts. Lastly, it was found that mothers’ self-reported insecurity was a risk factor for diarrhoea and dyspnea in children. The odds of diarrhoea and dyspnea in children increases with higher self-reported insecurity.

Self-reported insecurity of mothers was a protective factor for mother and child/children health, as mothers were more likely to adopt ANC and EIB when their level of insecurity increases. Armed conflict can provide improved access to health care, as residents in areas with severe conflicts are often relocated to government recognized internally-displaced persons (IDP) camps [25]. A study also pointed out that determinants of maternal healthcare utilisation by mothers include mothers’ fear of pregnancy-related complications, the empowerment of women, the support of females at family and community levels, the cost of services, and the proximity to health facilities [25]. Another study identified several barriers to maternal health service utilisation, which included financial, psychological, and socio-cultural barriers [26].

The positive association between mothers’ self-reported insecurity and maternal health-seeking behaviours in the DRC can be attributed to the availability and accessibility of maternal health services and mothers’ fear of pregnancy-related complications. For example, due to the severe conflicts in the Kasai region, the region was brought to the attention of local and international governmental agencies. The increased attention resulted in an influx of humanitarian aid by the DRC government, the U.S. Agency of International Development and UNICEF, to protect and improve the health status of vulnerable groups, which included women, newborns, and children under five living in the region [27,28]. Aids often include medical services such as maternal and child healthcare, allowing mothers living in the regions access to healthcare, thereby increasing the use of maternal and child health services. Another factor contributing to the higher use of maternal and child health services in conflict regions are the higher risks of adverse events posed to pregnant women living in conflict-affected regions [25].

The second finding could also be explained with the same reasons as mentioned above, as mothers living in regions with severe conflict were more likely to adopt ANC, EIB, and have skilled attendants during delivery than those living in regions with minor conflicts. This also relates to the availability of maternal and child health services and mothers’ fear of pregnancy-related complications.

The last finding indicates that higher self-reported insecurity of mothers was positively associated with diarrhoea and dyspnea in children under five. A previous study showed that conflicts result in inadequate and unsafe living conditions, produces environmental hazards, affect caregivers’ mental health, result in family separation, and increases health risks associated with displacement [29]. It was also reported that children living in conflict-affected environments have higher mortality [30]. This suggests that children are affected by conflict in many aspects including their living environment and health. Poor health in children (having diarrhoea, dyspnea etc.) can be attributed to having no professional medical advice, poor postpartum services, and low household status, which are common factors in conflict-affected regions [31]. The increasing levels of the severity of the conflict will exacerbate these factors, and therefore, increase the prevalence of childhood diseases.

This study was the first study to explore the association between self-reported insecurity with maternal and child health service utilisation in the DRC. Particularly, we combined two datasets from MICS6 and UCDP to demonstrate how the severity of conflicts impact maternal health-seeking behaviours in women with various socioeconomic status and demographic traits in the DRC. The findings of this study provide evidence-based information to better inform existing and future health policies in the DRC for women and children under five.

There are several limitations to this study. Firstly, only three levels of self-reported insecurity were used as exposures in the analyses. Hence, the specific degree of participants’ insecurities was not known. Secondly, data on conflicts and BRD could be incomplete as some were not registered by the UCDP. Thirdly, as this was a cross-sectional study, causality cannot be inferred between conflicts and maternal and child healthcare utilisation in the DRC.

## 5. Conclusions

Based on the analysis of MICS6 data in the DRC, we found an opposite association between conflicts and maternal health utilisation compared to previous studies. Our results showed that females with higher self-reported insecurity were more inclined to seek medical professionals in the Congolese health sectors. Additionally, we found that self-reported insecurity was a risk factor for diseases in children under five in the DRC. This association indicates that conflicts play an essential role in maternal and child health utilisation in the DRC. Additional health services with better standards need to be provided to mothers in both conflict and non-conflict areas to further improve maternal and child health conditions in the DRC.

## Figures and Tables

**Table 1:** Population and the incidence of battle-related deaths (BRD) of each province in the DRC (2016–2018).

Province	Population ^a^	Cumulative BRD Number	BRD Incidence (per Million People)
Kasai	2,313,020	2655	1147.85
Kasai Central	2,870,553	2237	779.29
Tanganyika	1,639,923	920	561
Maindombe	1,899,673	875	460.61
Nord Kivu	5,555,285	2516	452.9
Ituri	4,275,675	806	188.51
Sud Kivu	4,817,219	526	109.19
Maniema	1,946,690	87	44.69
Kasai Oriental	3,000,730	112	37.32
Lomami	2,146,227	42	19.57
Lualaba	2,140,924	38	17.75
Haut Uele	1,443,995	25	17.31
Haut-Katanga	4,382,428	62	14.15
Kinshasa	8,808,080	108	12.26
Kongo Central	4,624,229	39	8.43
Bas Uele	883,848	7	7.92
Sankuru	1,285,635	1	0.78
Equateur	1,806,970	1	0.55
Haut Lomami	2,526,306	1	0.4
Tshuapa	1,113,011	0	0
Mongala	1,227,929	0	0
Nord Ubangi	1,178,158	0	0
Sud Ubangi	2,016,644	0	0
Kwango	2,063,644	0	0
Kwilu	4,128,694	0	0
Tshopo	1,446,446	0	0

^a.^ Based on population estimates from the 2015 MICS in the DRC.

**Table 2:** Characteristics of females aged 15–49 with children under five †.

	Total	Low Insecurity	Medium Insecurity	High Insecurity	*p*-Value
Number of observations (N)	8144	5224	1933	987	
Percentage (%)	100	59.12	28.62	12.26	
Maternal age, year (SD)	1.66 (0.47)	1.69 (0.46)	1.59 (0.49)	1.63 (0.48)	0.0463
Region					<0.001
Urban	34.28	30.77	40.55	36.60	
Rural	65.72	69.23	59.45	63.40	
Household wealth					0.0658
Highest	11.30	10.68	11.86	12.92	
Upper middle	18.89	16.97	22.97	18.59	
Middle	20.71	21.85	19.26	18.67	
Lower middle	23.12	23.25	22.63	23.60	
Lowest	25.98	27.25	23.28	26.22	
Maternal education					0.0595
Preschool	18.74	18.48	17.72	22.40	
Primary school	35.98	35.22	38.53	33.73	
Lower secondary school	14.59	16.21	12.64	11.35	
Higher secondary school	27.68	27.69	27.03	29.19	
Above	3.00	2.41	4.08	3.33	
The severity of conflicts					<0.001
Low	37.25	46.51	22.66	26.65	
High	62.75	53.49	77.34	73.35	
Maternal health-seeking behaviours					
Skilled Antenatal Care (ANC) ≥ 4	41.52	37.31	44.86	54.06	<0.001
Skilled attendants at delivery	84.26	82.15	87.23	87.53	<0.001
Received Postnatal Care (PNC)	6.56	6.35	8.13	3.94	0.0245
Early initiation of breastfeeding (EIB)	47.41	44.93	50.03	53.26	0.0083

† All proportions are weighted.

**Table 3:** Characteristics of children under five †.

	Total	Low Insecurity	Medium Insecurity	High Insecurity	*p*-Value
Number of observations (N)	14,403	9245	3418	1740	
Percentage (%)	100	59.04	28.84	12.12	
Maternal age, year (SD)	29.14 (6.68)	29.30 (6.70)	29.09 (6.70)	28.51 (6.45)	<0.001
Region					<0.001
Urban	34.07	30.93	39.82	35.67	
Rural	65.93	69.07	60.18	64.33	
Household wealth					0.0018
Highest	11.11	10.32	11.96	12.98	
Upper middle	19.22	17.63	22.72	18.68	
Middle	20.84	21.93	19.7	18.21	
Lower middle	23.08	23.06	22.83	23.71	
Lowest	25.75	27.06	22.79	26.42	
Maternal education					<0.001
Preschool	19.27	19.34	17.26	23.68	
Primary school	36.59	35.43	39.86	34.46	
Lower secondary school	14.91	16.74	12.63	11.43	
Higher secondary school	26.34	26.08	26.28	27.76	
Above	2.89	2.41	3.97	2.67	
Child age, year (SD)	1.61 (1.43)	1.60 (1.43)	1.60 (1.43)	1.63 (1.47)	0.8830
Child sex					0.6406
Male	49.33	49.95	48.35	48.62	
Female	50.67	50.05	51.65	51.38	
The severity of conflicts					<0.001
Low	35.55	44.53	21.27	25.74	
High	64.45	55.47	78.73	74.26	
Child diseases					
Diarrhoea	15.75	13.35	19.32	18.97	<0.001
Fever	28.01	27.06	28.69	30.98	0.1420
Cough	26.33	24.41	27.58	32.72	<0.001
Dyspnea	9.36	7.07	12.15	13.92	<0.001

† All the proportions are weighted.

**Table 4:** Associations between self-reported insecurity with maternal health-seeking behaviours †.

	Low Insecurity	Medium Insecurity OR (95%CI)	High Insecurity OR (95%CI)
Skilled antenatal care (ANC) ≥4	[1]	1.30 (1.07, 1.58) **	1.93 (1.50, 2.49) ***
Skilled attendants at delivery		1.28 (1.02, 1.62)*	1.42 (1.08, 1.87) *
Received postnatal care (PNC)		1.55 (1.12. 2.16) **	0.69 (0.38, 1.24)
Early initiation of breastfeeding (EIB)		1.15 (0.96, 1.38)	1.32 (1.04, 1.68) *

† Adjusted for maternal age, region(rural/urban), household economic status, maternal education level, the severity of conflicts. * *p* < 0.05, ** *p* < 0.01, *** *p* < 0.001.

**Table 5:** Associations between self-reported insecurity with diseases in children under five †.

	Low Insecurity	Medium Insecurity OR (95%CI)	High Insecurity OR (95%CI)
Diseases in children under five			
Diarrhea	[1]	1.45 (1.21, 1.74) ***	1.47 (1.14, 1.88) **
Fever		1.08 (0.93, 1.25)	1.23 (1.01, 1.50) *
Cough		1.11 (0.95, 1.28)	1.45 (1.19, 1.77) ***
Dyspnea		1.68 (1.36, 2.08) ***	2.04 (1.52, 2.73) ***

† Adjusted for maternal age, region(rural/urban), household economic status, maternal education level, child age, child sex, the severity of conflicts. * *p* < 0.05, ** *p* < 0.01, *** *p* < 0.001.

**Table 6:** Associations between self-reported insecurity with maternal health-seeking behaviours, stratified by the severity of conflicts †.

	Low Insecurity		Medium Insecurity		High Insecurity	
	N	OR (95%CI)	N	OR (95%CI)	N	OR (95%CI)
Low severity of conflicts	2732		743		452	
Skilled antenatal care (ANC) ≥4	899	[1]	281	1.10 (0.81, 1.49)	164	1.24 (0.81, 1.88)
Skilled attendants at delivery	1853		470	0.67 (0.50, 0.89) **	268	0.83 (0.61, 1.14)
Received postnatal care (PNC)	239		101	2.30 (1.51, 3.50) ***	41	1.16 (0.50, 2.69)
Early initiation of breastfeeding (EIB)	1205		256	0.84 (0.63, 1.12)	170	0.82 (0.55, 1.22)
High severity of conflicts	2492		1190		535	
Skilled antenatal care (ANC) ≥4	905	[1]	493	1.40 (1.10, 1.77) **	248	2.23 (1.64, 3.03) ***
Skilled attendants at delivery	1980		1006	1.77 (1.29, 2.43) ***	448	1.91 (1.29, 2.82) **
Received postnatal care (PNC)	118		72	1.06 (0.67, 1.68)	19	0.39 (0.19, 0.77) **
Early initiation of breastfeeding (EIB)	1164		625	1.32 (1.05, 1.65) *	314	1.63 (1.21, 2.21) **

† Adjusted for maternal age, region(rural/urban), household economic status, maternal education level. * *p* < 0.05, ** *p* < 0.01, *** *p* < 0.001.

**Table 7:** Associations between self-reported insecurity with diseases in children, stratified by the severity of conflicts †.

	Low Insecurity		Medium Insecurity		High Insecurity	
	N	OR (95%CI)	N	OR (95%CI)	N	OR (95%CI)
Low severity of conflicts	4727		1272		770	
Diarrhea	644	[1]	223	1.56 (1.14, 2.14) **	135	1.40 (0.96, 2.02)
Fever	1183		413	1.27 (1.01, 1.61) *	235	1.18 (0.86, 1.64)
Cough	1030		364	1.40 (1,10, 1,77) **	211	1.29 (0.92, 1.81)
Dyspnea	334		138	1.64 (1.17, 2.29) **	88	1.91 (1.23, 2.95) **
High severity of conflicts	4518		2146		970	
Diarrhea	659	[1]	390	1.41 (1.13, 1.75) **	174	1.44 (1.06, 1.95) *
Fever	1259		605	1.01 (0.84, 1.22)	309	1.19 (0.93, 1.53)
Cough	1161		588	1.02 (0.85,1.23)	336	1.47 (1.15, 1.87) **
Dyspnea	354		243	1.70 (1.31, 2.21) ***	126	2.05 (1.44, 2.93) ***

† Adjusted for maternal age, region(rural/urban), household economic status, maternal education level, child age, child sex. * *p* < 0.05, ** *p* < 0.01, *** *p* < 0.001.

## Data Availability

This study used data from the Multiple Indicator Cluster Surveys (MICS) carried out in the DRC in 2018, a nationally representative household survey of children aged from 0–5, females aged from 15–49, and males aged from 15–59. The dataset can be found publicly (http://mics.unicef.org/surveys, accessed on 29 June 2021). The other dataset used in this study was UCDP Georeferenced Event Dataset (GED) Global version 20.1, which are openly available in The Uppsala Conflict Data Program at https://ucdp.uu.se/downloads/ (accessed on 29 June 2021) [21,22].

## Abbreviations

DRC	Democratic Republic of Congo
MICS	Multiple Indicators Cluster Survey
UCDP	Uppsala Conflict Data Program
UNICEF	United Nations Children’s Fund
MCH	Maternal and child health
SDGs	Sustainable Development Goals
UN	The United Nations
LMICs	Low- and middle-income countries
MMR	Maternal mortality ratio
ANC	Antenatal care
PNC	Post-natal care
BBC	British Broadcasting Corporation
USAID	United States Agency for International Development
GED	Georeferenced Event Dataset
BRDs	Battled related deaths
EIB	Early initiation breastfeeding
WHO	World Health Organisation
ORs	Odds ratios
CI	Confidence intervals
SD	Standard deviation
